# Supplementary material for: End Sequence Analysis Toolkit (ESAT) expands the extractable information from single-cell RNA-seq data
Source: Genome Res. 2016 Oct;26(10):1397–410. doi: 10.1101/gr.207902.116 (PMC5052061; doi:10.1101/gr.207902.116)
Supplement: Supplemental Material [file supp_gr.207902.116_Supp_Materials.tar.gz › ESAT-esat_v0.1/doc/html/classbroad_1_1pda_1_1gene_1_1functional_annotation.html]

ESAT: broad.pda.gene.functionalAnnotation Class Reference


ESAT


- Main Page
- Related Pages
- Packages
- Classes
- Files

- Class List
- Class Index
- Class Hierarchy
- Class Members

All Classes Namespaces Files Functions Variables Enumerator Pages

- broad
- pda
- gene
- functionalAnnotation

Public Member Functions |
Static Public Member Functions |
List of all members

broad.pda.gene.functionalAnnotation Class Reference

Collaboration diagram for broad.pda.gene.functionalAnnotation:

[legend]

|  |  |
| --- | --- |
| Public Member Functions | |
|  | functionalAnnotation () |
|  | |
| void | loadGoMap (String gofile, String goformat, int goSizeThreshold) throws IOException |
|  | |
| void | calcAllTermsEnrichment (Collection< Gene > geneSet, LinkedList< Collection< Gene >> randomGeneSets) |
|  | |
| void | calcTermEnrichment (String term, HashMap< String, String > geneSet, LinkedList< HashMap< String, String >> randomSets, Double[] permPval, Double[] fischerExact, Integer[] numOverlap, LinkedList< Double > randomFischerExact, LinkedList< Integer > randomOverlap) |
|  | |
| void | writeTermsEnrichments (String filename, int sizeThreshold, String str, ArrayList< String > termsSorted) throws IOException |
|  | |
| Set< Gene > | getTermGenes (String goTerm, BEDFileParser ref) |
|  | |

|  |  |
| --- | --- |
| Static Public Member Functions | |
| static void | parseGoRawFiles (String Ontology, String Annotation, String pid2gid, String nm2pidF, int nmCol, int pidCol, String outf) throws IOException |
|  | |
| static void | main (String[] args) throws Exception |
|  | |

## Constructor & Destructor Documentation

|  |  |  |  |  |
| --- | --- | --- | --- | --- |
| broad.pda.gene.functionalAnnotation.functionalAnnotation | ( |  | ) |  |

Here is the caller graph for this function:

## Member Function Documentation

|  |  |  |  |
| --- | --- | --- | --- |
| void broad.pda.gene.functionalAnnotation.calcAllTermsEnrichment | ( | Collection< Gene > | *geneSet*, |
|  |  | LinkedList< Collection< Gene >> | *randomGeneSets* |
|  | ) |  |  |

Here is the call graph for this function:

|  |  |  |  |
| --- | --- | --- | --- |
| void broad.pda.gene.functionalAnnotation.calcTermEnrichment | ( | String | *term*, |
|  |  | HashMap< String, String > | *geneSet*, |
|  |  | LinkedList< HashMap< String, String >> | *randomSets*, |
|  |  | Double[] | *permPval*, |
|  |  | Double[] | *fischerExact*, |
|  |  | Integer[] | *numOverlap*, |
|  |  | LinkedList< Double > | *randomFischerExact*, |
|  |  | LinkedList< Integer > | *randomOverlap* |
|  | ) |  |  |

Here is the caller graph for this function:

|  |  |  |  |
| --- | --- | --- | --- |
| Set<Gene> broad.pda.gene.functionalAnnotation.getTermGenes | ( | String | *goTerm*, |
|  |  | BEDFileParser | *ref* |
|  | ) |  |  |

|  |  |  |  |
| --- | --- | --- | --- |
| void broad.pda.gene.functionalAnnotation.loadGoMap | ( | String | *gofile*, |
|  |  | String | *goformat*, |
|  |  | int | *goSizeThreshold* |
|  | ) |  | throws IOException |

|  |  |  |  |  |  |
| --- | --- | --- | --- | --- | --- |
| static void broad.pda.gene.functionalAnnotation.main | ( | String[] | *args* | ) | throws Exception |

static

Here is the call graph for this function:

|  |  |  |  |
| --- | --- | --- | --- |
| static void broad.pda.gene.functionalAnnotation.parseGoRawFiles | ( | String | *Ontology*, |
|  |  | String | *Annotation*, |
|  |  | String | *pid2gid*, |
|  |  | String | *nm2pidF*, |
|  |  | int | *nmCol*, |
|  |  | int | *pidCol*, |
|  |  | String | *outf* |
|  | ) |  | throws IOException |

static

Here is the caller graph for this function:

|  |  |  |  |
| --- | --- | --- | --- |
| void broad.pda.gene.functionalAnnotation.writeTermsEnrichments | ( | String | *filename*, |
|  |  | int | *sizeThreshold*, |
|  |  | String | *str*, |
|  |  | ArrayList< String > | *termsSorted* |
|  | ) |  | throws IOException |

---

The documentation for this class was generated from the following file:

- java/broad/pda/gene/functionalAnnotation.java


---

Generated on Fri Aug 15 2014 15:10:59 for ESAT by  

 1.8.7
